# miR-106b-5p as a Central Regulator of Cancer Progression and Chemotherapy-Induced Cardiotoxicity: From Molecular Mechanisms to Clinical Translation

**DOI:** 10.3390/ijms262010002

**Published:** 2025-10-14

**Authors:** Maria del Carmen Asensio Lopez, Miriam Ruiz Ballester, Francisco Jose Bastida Nicolas, Fernando Soler Pardo, Jose Luis Alonso-Romero, Cesar Caro-Martinez, Domingo Pascual Figal, Antonio Lax

**Affiliations:** 1 R&D Department, Biocardio S.L., El Palmar, 30120 Murcia, Spain; 2 Instituto Murciano de Investigación Biosanitaria (IMIB) Pascual Parrilla, University of Murcia, 30120 Murcia, Spain; miriam.ruiz4@um.es (M.R.B.); fjose.bastida@um.es (F.J.B.N.); fsoler@um.es (F.S.P.); 3 Centro Nacional de Investigaciones Cardiovasculares (CNIC), 28029 Madrid, Spain; dpascual@um.es; 4 Servicio de Oncología Médica, Hospital Clínico Universitario Virgen de la Arrixaca, 30120 Murcia, Spain; josel.alonso2@carm.es; 5 Heart Failure Unit, Cardiology Department, Virgen de la Arrixaca Hospital, School of Medicine, University of Murcia, 30120 Murcia, Spain; ccaro1980@gmail.com; 6 Cardiology Department, Hospital Virgen de la Arrixaca, IMIB-Pascual Parrilla, University of Murcia, El Palmar, 30120 Murcia, Spain; 7 CIBER en Enfermedades Cardiovasculares (CIBER-CV), 28029 Madrid, Spain

**Keywords:** miR-106b-5p, cardiotoxicity, anthracyclines, cancer progression, OncomiR, heart failure, PP2A/HDAC4/YY1 signaling, antagomir therapy, cardio-oncology, precision medicine

## Abstract

MicroRNAs (miRNAs) are critical regulators of gene expression in cancer biology and cardiovascular disease. miR-106b-5p, a member of the miR-106b-25 cluster, has been widely studied for its oncogenic activity in various malignancies. However, its role as a direct molecular driver of anthracycline-induced cardiotoxicity has only recently been uncovered. This finding highlights new therapeutic possibilities at the intersection of oncology and cardiovascular medicine. This review outlines the dual role of miR-106b-5p as a key modulator in both tumor progression and chemotherapy-induced cardiac dysfunction. miR-106b-5p is upregulated in numerous cancers—including breast, prostate, lung, gastric, colorectal, hepatocellular, and esophageal—and promotes tumorigenesis via suppression of tumor suppressors such as PTEN, BTG3, p21, and SMAD7, leading to activation of oncogenic pathways like PI3K/AKT and TGF-β. Importantly, we present the first evidence that miR-106b-5p is significantly upregulated in the myocardium in response to doxorubicin treatment, where it drives left ventricular dysfunction by targeting PR55α, a key regulator of PP2A activity. This pathway results in cytoplasmic HDAC4 accumulation, aberrant activation of the YY1 transcription factor, and upregulation of sST2, a biomarker linked to adverse cardiac remodeling and poor prognosis. In response, we developed AM106, a novel locked nucleic acid antagomir that silences miR-106 b-5p. Preclinical studies demonstrate that AM106 restores PR55α/PP2A activity, reduces sST2 expression, and prevents structural and functional cardiac damage without compromising anti-tumor efficacy. In parallel, artificial intelligence (AI) tools could be leveraged in the future—based on established AI applications in miRNA cancer research—to accelerate the identification of miR-106b-5p-related biomarkers and guide personalized therapy selection. Our findings position miR-106b-5p as a previously unrecognized molecular bridge between cancer and doxorubicin-induced cardiotoxicity. The development of the AM106 antagomir represents a promising approach with potential clinical applicability in cardio-oncology, offering dual benefits: tumor control and cardioprotection. Coupling this innovation with AI-driven analysis of patient data may enable precision risk stratification, early intervention, and improved outcomes. miR-106b-5p thus emerges as a central therapeutic target and biomarker candidate for transforming the clinical management of cancer patients at risk for heart failure.

## 1. Introduction

MicroRNA (miRNA) molecules are small (20–24 nucleotides), non-coding RNA sequences that play a crucial role in regulating gene expression at the post-transcriptional level by affecting both mRNA stability and translation [1,2]. These molecules are involved in various essential biological processes, including cell proliferation, differentiation, apoptosis, and development [3]. Dysregulation of miRNAs has been implicated in numerous human diseases, including cancer and cardiovascular disorders, highlighting their role as master regulators with pleiotropic effects across multiple disease contexts [4,5].

In this context, miR-106b-5p, located on chromosome 7q22.1, emerges as a member of the highly conserved miR-106b-25 cluster, including miR-93 and miR-25 [6,7]. miR-106b and miR-93 share the same seed sequence, while miR-25 has a similar seed sequence, potentially leading to overlapping but also distinct mRNA target profiles. The miR-106b-25 cluster is within intron 13 of the minichromosome maintenance complex component 7 (MCM7) gene [8]. Their localization within an intron of MCM7, a gene involved in DNA replication, suggests potential co-regulation and a link to cell proliferation and the cell cycle, central processes in both cancer and cardiac remodeling. Research has implicated miR-106b-5p in various cancer types, acting as both an oncogene and a tumor suppressor, depending on the context [9,10,11,12]. Furthermore, its involvement in cardiovascular diseases, including cardiotoxicity and heart failure, has been recently recognized [13,14]. Given its dual role in cancer and cardiovascular disease, this review focuses on miR-106b-5p as a key molecular nexus underlying both tumor progression and cardiotoxicity, exploring overlapping target genes and pathways, and highlighting its potential for translational applications in diagnostics, prognostics, and therapeutics.

### 1.1. The Biogenesis of miR-106b-5p

The biogenesis of microRNAs (miRNAs) is a tightly regulated, multi-step process that couples transcriptional control to post-transcriptional processing and ultimately to effector loading into the RNA-induced silencing complex (RISC). The canonical pathway—transcription by RNA polymerase II, nuclear cropping by the Microprocessor complex (Drosha-DGCR8), nuclear export by Exportin-5, cytoplasmic cleavage by Dicer, and Argonaute (AGO) loading of the guide strand—remains the central framework for miRNA maturation and is well established in mammalian cells. This canonical description provides the baseline for understanding miR-106b-5p maturation and is supported by comprehensive reviews and mechanistic studies [15].

#### 1.1.1. Genomic Context and Transcriptional Regulation

miR-106b-5p is encoded within the miR-106b~25 cluster (miR-106b, miR-93 and miR-25), which is located in an intron of the minichromosome maintenance protein 7 (MCM7) gene. Consequently, the expression of miR-106b-5p is frequently co-regulated with MCM7 transcription, linking the miRNA’s expression to cell-cycle and proliferative transcriptional programs. Multiple studies indicate that transcription factors frequently implicated in cell cycle control and oncogenesis (notably E2F family members) drive MCM7/miR-106b~25 transcription, a mechanism that explains coordinated overexpression of the cluster in a variety of tumors.

#### 1.1.2. Nuclear Processing and Modulation by Accessory Factors

After transcription, the primary transcript (pri-miRNA) containing the miR-106b~25 hairpins is processed in the nucleus by Drosha (in complex with DGCR8) into precursor hairpins (pre-miRNAs). Recent work has highlighted that miRNA biogenesis is not solely dependent on the core enzymes (Drosha/DGCR8, Dicer, XPO5) but is dynamically modulated by RNA-binding proteins (RBPs) and by alternative splicing or host gene isoforms that can affect intronic miRNA maturation and abundance. Such RBPs (for example, hnRNPs, LIN28 family members and others) can enhance or inhibit cropping and thereby modulate mature miR-106b-5p levels in a context-dependent manner. These regulatory layers explain tissue-specific or stress-responsive changes in miR-106b-5p expression observed in recent studies [16].

#### 1.1.3. Cytoplasmic Processing, Strand Selection and RISC Loading

Following nuclear export by Exportin-5, pre-miR-106b hairpins are cleaved by Dicer to generate a ~21–24 nt duplex. Strand selection (5p vs. 3p) is influenced by thermodynamic stability at the duplex ends and by specific protein cofactors; in the case of the miR-106b~25-derived duplex, the -5p strand (miR-106b-5p) is the predominant guide strand loaded into AGO proteins in many cell types, although context-dependent variation in 5p/3p usage has been reported. Once loaded into RISC, miR-106b-5p directs post-transcriptional silencing via seed-mediated recognition of target mRNAs, resulting in translational repression and/or mRNA decay depending on complementarity and cellular context [15].

#### 1.1.4. Host Gene-Cluster Cooperation and Oncogenic Dysregulation

Functionally, the intronic location of the miR-106b~25 cluster within MCM7 has important biological implications. Preclinical and clinical data indicate cooperative oncogenic activity between MCM7 and its hosted miRNAs: overexpression of MCM7 is frequently accompanied by upregulation of miR-106b-5p and its sibling miRNAs, contributing to proliferation, evasion of apoptosis and therapeutic resistance across several cancer types. Recent large-scale and focused studies (including publications in 2024–2025) have reinforced the pathogenic significance of this host-cluster axis and provided mechanistic links to pathways such as TGF-β/SMAD regulation, YAP/TAZ signaling, and E2F-driven cell cycle programs. These findings underscore that alterations in host gene expression, splicing, or chromatin state can directly influence miR-106b-5p levels and thereby modulate oncogenic phenotypes [17].

#### 1.1.5. Contextual and Stress-Responsive Regulation—Implications for Experiments

Emerging evidence up to 2025 emphasizes that miR-106b-5p abundance is sensitive to cellular stressors (e.g., ER stress, DNA damage), host-gene splicing variants, and microenvironmental cues; these factors alter Drosha/DGCR8 or Dicer processing efficiency or the activity of RBPs that bind pri-/pre-miRNA structures. Therefore, when interpreting expression data or designing functional experiments for miR-106b-5p, investigators should control for host-gene expression, splice isoforms, RBP expression, and cellular stress status to avoid confounding. Additionally, measurements of both precursor and mature miRNA forms (and examination of 5p/3p ratios) improve mechanistic interpretation [18].

#### 1.1.6. Summary (Practical Takeaways)

**Figure 1:**
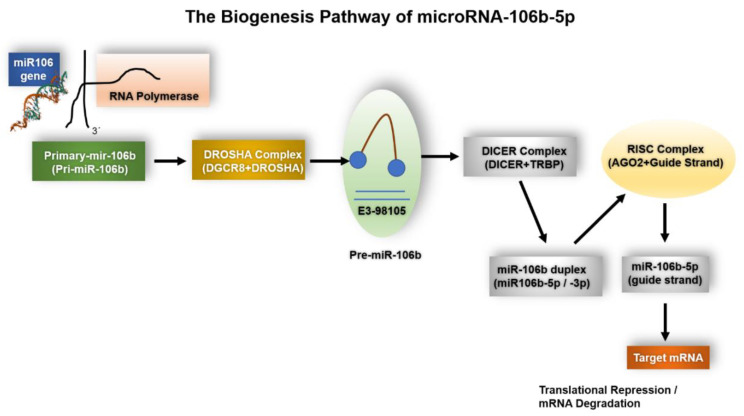
The Biogenesis Pathway of miR-106b-5p. Schematic representation of the canonical microRNA biogenesis pathway leading to the maturation of miR-106b-5p. The MIR106B gene is transcribed by RNA polymerase II into a primary transcript (pri-miR-106b), which is processed in the nucleus by the Drosha–DGCR8 microprocessor complex into a precursor hairpin (pre-miR-106b). The pre-miRNA is exported to the cytoplasm by Exportin-5 in a GTP-dependent manner and further cleaved by the Dicer–TRBP complex into a miRNA duplex. The guide strand (miR-106b-5p) is selectively incorporated into the Argonaute-containing RISC complex, while the passenger strand (miR-106b-5p) is degraded. Finally, miR-106b-5p directs translational repression or mRNA degradation of its target transcripts.

- miR-106b-5p arises from the intronic miR-106b~25 cluster within MCM7 and is commonly co-expressed with its host gene, a relationship with strong relevance for tumor biology [17].
- Canonical processing (Drosha → Exportin-5 → Dicer → AGO) describes the maturation route (Figure 1), but is modulated by RBPs, alternative splicing, and host-gene regulation—factors that account for tissue- and context-specific variations in mature miR-106b-5p levels [15].
- Experimental designs should monitor host-gene expression and precursor/mature ratios, and consider RBP and stress-response states, to draw robust conclusions about miR-106b-5p function [18].

## 2. Elevated miR-106b-5p Expression and Tumorigenic Roles in Specific Cancers

Briefly, miR-106b-5p exhibits elevated expression across a wide range of human cancers, playing diverse and often context-dependent roles in the development and progression of these diseases [19,20]. Its involvement in key oncogenic processes, including cell proliferation, inhibition of apoptosis, migration, invasion, metastasis, and regulation of the cell cycle, is mediated through the targeting of various tumor suppressor genes and oncogenes, such as RBL1, RBL2, CASP8, BTG3, FAT4, CTSA, PTEN, SMAD7, ALEX1, DLC-1, APC, and HPGD [7]. On the other hand, the observation that miR-106b-5p can exhibit opposing roles in certain cancers, such as breast and colorectal cancer, underscores the critical importance of considering the specific cellular and molecular environment when investigating the function of miRNAs. This duality is particularly evident in breast cancer, where the role of miR-106b-5p has been the subject of extensive investigation.

### 2.1. Breast Cancer: Examining the Dual Nature of miR-106b-5p

Elevated expression of miR-106b-5p has been consistently observed in breast cancer tissues, and this upregulation is often associated with the progression of the disease [19]. However, the precise role of miR-106b-5p in breast cancer appears to be complex and context-dependent, with some investigations suggesting it may even function as a tumor suppressor in specific scenarios [19]. For instance, one study indicated that miR-106b-5p is upregulated in aggressive subtypes of breast cancer, leading to the downregulation of genes such as GAB1, GNG12, HBP1, and SESN1, which are thought to have tumor-suppressing properties. This downregulation correlated with poorer survival outcomes for patients [21]. Conversely, in a model of ductal carcinoma in situ (DCIS), increased expression of miR-106b-5p, along with miR-17-5p, was found in recurrent cases [22]. These miRNAs activated the TGF-β pathway, potentially promoting tumor proliferation, illustrating the paradoxical effects of TGF-β in different stages of breast cancer development [23]. Furthermore, miR-106b-5p has been shown to contribute to the lung metastasis of breast cancer by targeting the CNN1 gene and subsequently regulating the Rho/ROCK1 signaling pathway [24]. The conflicting evidence regarding the role of miR-106b-5p in breast cancer, where it appears to act as both an oncogene and a potential tumor suppressor, underscores the critical influence of the cellular environment and the specific molecular context in determining miRNA function. Future research should prioritize identifying the subtypes, stages, or genetic backgrounds of breast cancer in which miR-106b-5p exhibits tumor-suppressive activity versus its more commonly reported oncogenic roles.

### 2.2. Prostate Cancer: Oncogenic Functions and Target Genes

In prostate cancer, miR-106b-5p contributes to tumor viability and migration, functioning as an oncogene [19]. Similar to its behavior in breast cancer, miR-106b-5p is often overexpressed in prostate cancer tissues and shows an inverse correlation with the expression of the tumor suppressor gene PTEN [19,25]. The expression levels of miR-106b-5p have been linked to tumor development and the recurrence of the disease through the targeting of genes such as Caspase-7 and those involved in focal adhesion, which are crucial for cell survival and metastasis [19]. Additionally, miR-106b-5p can influence the cellular response to radiation therapy by downregulating the p21 gene, leading to cell cycle arrest [19,26]. The consistent overexpression of miR-106b-5p and its inverse relationship with PTEN in both breast and prostate cancer suggest a conserved oncogenic mechanism. PTEN is a known tumor suppressor and a key negative regulator of the PI3K/Akt signaling pathway, which is frequently hyperactivated in cancer [27]. Therefore, miR-106b-5p likely promotes oncogenesis in these cancers by suppressing PTEN, resulting in increased PI3K/Akt signaling, thereby enhancing cell survival and proliferation. Further investigation into the precise regulatory mechanisms and the downstream effects on cell survival, proliferation, and metastasis in these cancers is warranted.

### 2.3. Lung Cancer (NSCLC): Promoting Proliferation and Inhibiting Apoptosis via BTG3

miR-106b-5p exhibits high expression levels in non-small cell lung cancer (NSCLC) cells [19]. It has been shown to enhance cell proliferation and suppress apoptosis by directly targeting BTG3, a gene that normally inhibits cell cycle progression and metastasis [19,28]. The overexpression of miR-106b-5p in NSCLC models has been demonstrated to promote tumor formation in vivo [29]. Interestingly, one study reported that miR-106b-5p inhibits the growth and progression of lung adenocarcinoma cells by downregulating IGSF10 [24,30], indicating that its role might vary depending on the specific histological subtype within lung cancer. Furthermore, miR-106-5p has been implicated in the resistance of NSCLC cells to cisplatin, a common chemotherapy drug, by directly targeting the PKD2 gene [19]. The identification of BTG3 as a key target of miR-106b-5p in promoting proliferation and inhibiting apoptosis in NSCLC offers a potential avenue for therapeutic intervention. Developing strategies to upregulate BTG3 expression or inhibit the activity of miR-106b-5p could represent novel treatment approaches for this aggressive form of cancer. The contrasting finding regarding IGSF10 highlights the heterogeneity within lung cancer and suggests that the role of miR-106b-5p might indeed differ between various histological subtypes, such as adenocarcinoma versus squamous cell carcinoma.

### 2.4. Gastric Cancer: Upregulation and Impact on Tumorigenesis

In gastric cancer, miR-106b-5p is significantly upregulated in both tumor tissues and the plasma of patients [19]. This elevated expression has been shown to alter the characteristics of cancer stem cells by directly targeting SMAD7 and consequently inhibiting the TGF-β signaling pathway in CD44-positive gastric cancer cells [19,31]. Moreover, miR-106b-5p expression in cancer-associated fibroblasts (CAFs) from gastric cancer is linked to a poorer prognosis, as it promotes cell migration and invasion through the regulation of PTEN [32]. Additionally, miR-106b-5p targets ALEX1, a known tumor suppressor gene, thereby promoting metastasis and inhibiting apoptosis in gastric cancer cells [19,33]. The miR-106b-25 cluster, particularly miR-106b, is upregulated in specific subsets of gastric tumors, and its expression correlates with aggressive clinicopathological features such as lymph node metastases, distant metastasis, and advanced TNM stage [34]. The involvement of miR-106b-5p in modulating both cancer stem cells, via SMAD7, and the tumor microenvironment, through PTEN regulation in CAFs, highlights its broad impact on gastric cancer tumorigenesis. Targeting miR-106b-5p could potentially disrupt multiple critical aspects of cancer progression in this disease.

### 2.5. Colorectal Cancer: Context-Dependent Effects on Migration and Invasion, Targeting FAT4 and CTSA

miR-106b-5p is significantly overexpressed in tissues of metastatic colorectal cancer [19]. It has been shown to influence the migration and invasion of colorectal cancer cells by targeting FAT4, with overexpression of miR-106b-5p promoting these processes [35]. However, some studies suggest that miR-106b-5p may inhibit invasion and metastasis by binding to CTSA (cathepsin A), indicating a role that is dependent on the specific context [35,36]. The metastatic suppressor gene DLC-1 is also a direct target of miR-106b-5p, and its downregulation by the miRNA enhances cell migration and invasion in colorectal cancer [19]. Furthermore, miR-106b-5p has been implicated in the radioresistance of colorectal cancer cells through its direct targets PTEN and p21.1 [26]. The opposing effects of miR-106b-5p on migration and invasion in colorectal cancer, depending on whether it targets FAT4 or CTSA, underscore the complexity of miRNA function and the possibility that different regulatory networks become dominant in different stages or subtypes of colorectal cancer. Further research is necessary to clarify the factors that determine which target is preferentially regulated by miR-106b-5p in this disease.

### 2.6. Hepatocellular Carcinoma: Driving Proliferation, Migration, and Cell Cycle Progression Through PTEN/PI3K/Akt

In hepatocellular carcinoma (HCC), miR-106b-5p expression is frequently increased and is associated with tumor progression, poorer survival rates, and an increased likelihood of recurrence in patients with HBV-associated HCC [19]. Notably, patients with well-differentiated HCC tend to show lower levels of miR-106b expression compared to those with moderately and poorly differentiated tumors, suggesting a role in differentiation and survival [19]. Overexpression of miR-106b-5p has been shown to upregulate Cyclin D1 by downregulating APC, thereby controlling the entry of cells into the G1/S phase of the cell cycle [19,37]. Additionally, Smad-7 is a target of miR-106b-5p, and its regulation of TGF-β1 and p-Smad3 results in miR-106b-5p promoting cell growth, migration, and the epithelial–mesenchymal transition (EMT) by suppressing the expression of E-cadherin [19]. RhoGTPases are also indirect targets of miR-106b-5p, influencing stress fiber formation and cell migration [38]. Targeting miR-106b-5p has been shown to help overcome TRAIL tolerance by increasing the expression of DR4.1. Zbtb7a is another target of miR-106b-5p, and its suppression inhibits apoptosis in HCC cells [19]. Furthermore, miR-106-5p expression is increased in HCC, exhibiting cancer stem cell properties and regulating cell migration through PTEN via the PI3K/AKT pathway [39]. The consistent finding that miR-106b-5p targets PTEN and activates the PI3K/Akt pathway in HCC strongly indicates that this is a central mechanism driving the progression of this cancer. Inhibiting miR-106b-5p or targeting downstream components of the PI3K/Akt pathway could, therefore, represent effective therapeutic strategies for HCC. The involvement of miR-106b-5p in the G1/S transition further emphasizes its role in promoting uncontrolled cell proliferation, a hallmark of cancer.

### 2.7. Esophageal Squamous Cell Carcinomas: Contribution to Tumorigenesis

In esophageal squamous cell carcinoma (ESCC), miR-106b-5p acts as an oncogene, contributing to proliferation, invasion, and the formation of metastases [19]. Its expression levels are typically elevated in ESCC tissues. miR-106b-5p directly targets PTEN and is involved in invasion, metastasis, and proliferation; notably, the downregulation of PTEN can rescue the induction of EMT [19]. Increased expression of miR-106b-5p is also implicated in lymph node metastases and promotes cell invasion and migration through EMT by downregulating SMAD-7 [19]. Additionally, miR-106b-5p targets HPGD, promoting proliferation, migration, and invasion and inhibiting apoptosis in ESCC cells [40]. Interestingly, one study reported that miR-106b expression was significantly decreased in ESCC serum samples compared to controls [41], suggesting a potential discrepancy between tissue and circulating levels of the miRNA. The consistent upregulation of miR-106b-5p in ESCC tissues and its promotion of key oncogenic processes such as proliferation, invasion, and metastasis through multiple targets (PTEN, SMAD-7, HPGD) make it a promising target for therapeutic intervention in ESCC. However, the observed difference in serum levels warrants further investigation to determine its potential as a diagnostic biomarker for this disease.

### 2.8. miR-106b-5p: A Key Driver of Renal Cell Carcinoma Aggressiveness via Wnt/β-Catenin Signaling

miR-106b-5p has emerged as a consistent pro-oncogenic effector in clear-cell renal cell carcinoma (ccRCC), with multiple mechanistic studies (2015–2019) and more recent biomarker-oriented reviews (2024–2025) converging on its role in promoting aggressiveness and stem-like behavior. In a landmark functional study, Liu et al. demonstrated that forced expression of miR-106b-5p in ccRCC cell lines increases sphere-forming capacity, expands the Hoechst-excluding side-population, and upregulates canonical stemness markers (SOX2, OCT4, ABCG2, CXCR4), concomitant with enhanced nuclear translocation of β-catenin and induction of downstream Wnt targets (CCND1, MYC, CD44, MMP7), whereas miR-106b-5p knockdown reverses these phenotypes—supporting a model whereby miR-106b-5p drives stemness through constitutive activation of the Wnt/β-catenin axis [42]. Complementary mechanistic work has shown that miR-106b-5p represses tumor-suppressive nodes beyond Wnt signaling: Xiang and colleagues reported post-transcriptional inactivation of the histone methyltransferase SETD2 in ccRCC Via direct targeting by miR-106b-5p, providing an epigenetic route to genomic instability and malignant progression [43]. Moreover, Miao et al. identified Capicua (CIC) as an additional miR-106b target that modulates MAPK pathway activity and thereby promotes proliferation and invasion in renal carcinoma models, illustrating that miR-106b-5p operates through multiple, partially non-redundant oncogenic circuits [44]. Translationally, these mechanistic data are paralleled by growing interest in circulating and exosomal miR-106b species as diagnostic/prognostic analytes in RCC: recent reviews and translational reports highlight panels that include miR-106b and note its recurrent upregulation across tissue and biofluid studies, supporting its candidacy for non-invasive detection while also underscoring heterogeneity in assay platforms and study cohorts [19,35]. Taken together, the body of evidence through 2025 supports a model in which miR-106b-5p functions as a multifunctional driver of ccRCC aggressiveness—activating Wnt/β-catenin signaling, disabling epigenetic safeguards, and engaging MAPK-linked effectors—yet important gaps remain (standardization of biofluid assays, large prospective validation of prognostic value, and in-depth mapping of upstream regulators such as lncRNAs and transcriptional control), all of which must be addressed before miR-106b-5p-directed interventions can be credibly advanced toward the clinic.

### 2.9. miR-106b-5p in Bladder Cancer

Accumulating evidence over the past five years supports a nuanced role for miR-106b-5p in bladder cancer biology and prognosis, particularly within molecularly defined p53-like subtypes. Early functional work (Lee et al.) identified miR-106b activity as a discriminator of p53-like bladder tumors with differing prognoses; subsequent translational studies have focused on validating miR-106b and miRNA panels in tissue and biofluids for diagnostic and prognostic use [11,45]. Systematic reviews and biomarker surveys published in 2022–2024 show that miR-106b-5p frequently appears in multi-marker signatures that distinguish muscle-invasive from non-muscle-invasive disease and that correlate with recurrence risk, although findings are heterogeneous across cohorts and platforms [46,47]. Mechanistically, recent computational and wet-lab studies have linked miR-106b-5p activity to cell cycle regulators and pathways involved in DNA damage response and apoptosis—consistent with its context-dependent tumor-suppressor or oncogenic behavior observed in earlier reports—suggesting that its prognostic value may depend on coexisting molecular features (e.g., TP53 status, transcriptomic subtype) and tumor microenvironmental cues [45,46]. Importantly, advances from 2020 to 2024 emphasize translational barriers: variance in sample preparation (urine vs. plasma), normalization strategies, and small, retrospective cohorts limit reproducibility. Larger prospective cohorts with standardized extraction/assay pipelines and orthogonal validation (e.g., exosomal vs. cell-free miRNA) are therefore necessary to determine whether miR-106b-5p can be clinically deployed as a single marker or should remain part of multi-analyte panels for risk stratification and therapy selection in bladder cancer [46,47].

### 2.10. miR-106b-5p in Cutaneous Melanoma

From 2020 to 2025, the literature has consolidated miR-106b-5p as a pro-tumorigenic miRNA in cutaneous melanoma with both cell-intrinsic and microenvironmental (exosomal) actions. Luan et al. (2021) provided direct mechanistic evidence that melanoma cells secrete exosomes enriched in miR-106b-5p, which are taken up by neighboring melanocytes; exosomal miR-106b-5p represses EphA4 and thereby activates ERK signaling to induce epithelial-to-mesenchymal transition (EMT), migration, and invasion—phenotypes linked to metastasis and poor outcome [48]. This exosome-centric paradigm has been reinforced by 2022–2024 reviews and experimental studies that document elevated circulating and exosomal miR-106b-5p in melanoma patients and associate high expression with advanced stage and worse overall survival, implicating miR-106b-5p as both a mechanistic driver and a candidate circulating biomarker [49,50]. While earlier reports proposed PTEN as an intracellular target mediating cell-cycle effects, caution is warranted because some original PTEN-targeting data have been questioned; contemporary studies therefore focus on the robust exosome→EphA4→ERK axis and on profiling studies that replicate miR-106b-5p upregulation across independent cohorts [48,50]. Therapeutically, preclinical work suggests that blocking exosome release or neutralizing exosomal miR-106b-5p reduces phenotype switching and metastatic traits in model systems, but clinical translation remains nascent and will require standardized exosome assays and safety data for nucleic-acid-targeting approaches [49]. Collectively, work through 2025 positions miR-106b-5p as a multifunctional effector in melanoma progression—one whose extracellular trafficking via exosomes is central to its pathogenicity and biomarker potential.

**Figure 2:**
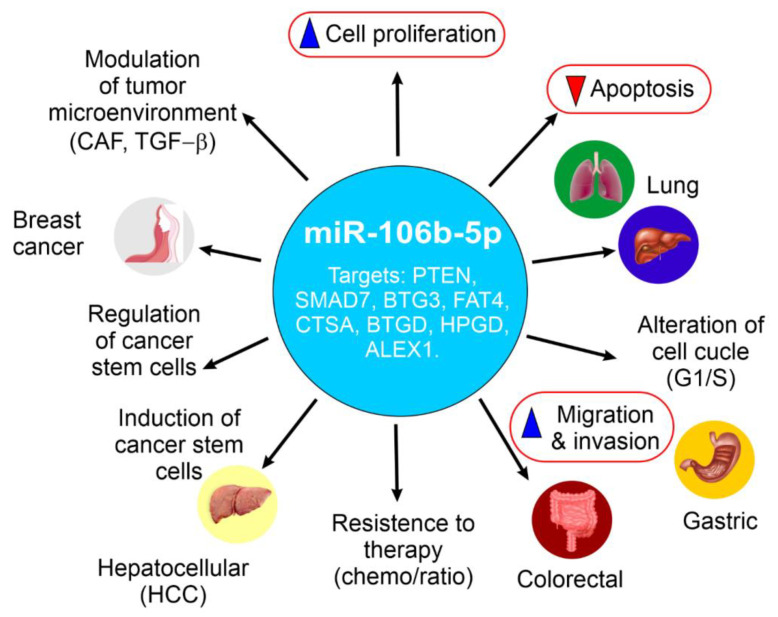
Overview of the oncogenic and context-dependent roles of miR-106b-5p in various human cancers. This schematic summarizes the expression patterns and functional roles of miR-106b-5p across several cancer types, including breast, prostate, lung (NSCLC), gastric, colorectal, hepatocellular carcinoma (HCC), and esophageal squamous cell carcinoma (ESCC). miR-106b-5p is frequently upregulated and promotes tumor progression by targeting key tumor suppressor genes such as PTEN, SMAD7, APC, BTG3, FAT4, and HPGD, contributing to processes such as proliferation, migration, invasion, metastasis, cell cycle progression, and therapy resistance. Notably, miR-106b-5p exhibits dual or context-dependent roles in breast and colorectal cancer, where it may act as either an oncogene or tumor suppressor depending on cellular context, molecular subtype, or disease stage. This highlights the importance of considering the tumor microenvironment and genetic landscape when evaluating miR-106b-5p as a potential biomarker or therapeutic target. This figure has been created by MCAL and AL using CorelDRAW X8. Data and materials are available at the UMU Institutional Repository DIGITUM, which is protected by copyright Creative Commons.

Figure 2 provides a schematic summary of miR-106b-5p expression patterns and its functional roles across breast, prostate, lung (NSCLC), gastric, colorectal, hepatocellular carcinoma (HCC), and esophageal squamous cell carcinoma (ESCC), highlighting its oncogenic and context-dependent behavior.

The schematic overview above is further detailed in the following comparative Table 1, which summarizes the specific roles of miR-106b-5p in various cancers, including key target genes and relevant signaling pathways.

## 3. Integrating Artificial Intelligence into miR-106b-5p Cancer Research

Artificial intelligence (AI) tools, particularly machine learning algorithms, have become increasingly essential in cancer research due to their ability to identify intricate patterns within large-scale omics datasets, including gene expression data and microRNA profiles [76]. AI’s capacity to analyze vast quantities of data allows for the identification of correlations between gene or miRNA expression levels and various clinical outcomes, such as patient survival, treatment response, and disease recurrence. This capability aids in patient stratification and the development of personalized treatment strategies [76]. For example, machine learning methods like CancerSig have been developed to pinpoint cancer stage-specific miRNA signatures for early cancer predictions by analyzing miRNA expression profiles across numerous cancer types [76]. AI is also being utilized to create diagnostic models for different cancers based on circulating miRNA profiles in the blood, potentially offering less invasive liquid biopsy approaches for early detection. Furthermore, AI-powered computational programs can even predict the activity of thousands of genes within tumor cells using only standard microscopy images of biopsies, potentially providing valuable gene expression information without the need for costly genomic sequencing.

Bioinformatic tools and databases complement AI approaches by providing validated and predicted information on miRNAs, their targets, regulatory networks, and expression across tissues and cancers. Databases such as miRTarBase offer experimentally validated microRNA-target interactions (MTIs), compiled from reporter assays, Western blots, microarrays, and NGS experiments [77]. Tools like TargetScan, miRDB, DIANA Tools (microT-CDS, TarBase, LncBase), miRWalk, miRPathDB, StarBase, and MirGeneDB supply both predictive target sites and functional annotation, and are widely used for hypothesis generation [78].

For example, miRnalyze integrates TargetScan predictions with signaling pathway databases such as KEGG, helping researchers to identify miRNAs that regulate entire pathways rather than single genes. This can help focus on biologically relevant interactions [79].

Furthermore, databases like CancerMIRNome integrate extensive datasets of miRNA expression profiles from the Cancer Genome Atlas (TCGA) and other public sources, incorporating AI tools for pan-cancer analysis and the discovery of potential biomarkers [80]. The ability of AI to process and analyze high-dimensional omics data far exceeds human capabilities, making it an invaluable tool for uncovering complex relationships between miRNA expression and cancer characteristics. This can lead to the discovery of novel biomarkers for diagnosis, prognosis, and the prediction of treatment response.

### 3.1. Limited Specific AI Studies on miR-106b-5p: Opportunities for Future Research

While AI is being increasingly employed in cancer research for general miRNA analysis, current studies specifically using AI or advanced bioinformatics to examine miR-106b-5p are still relatively few. One relevant bioinformatic study, *MiRNA-mRNA correlations in pancreatic cancer*, reported that hsa-miR-106b-5p showed the highest number of negative correlations between predicted targets and mRNA expression levels, highlighting its potential regulatory roles [81]. Another recent predictive tool, mintRULS, identifies several strong-target gene pairs for hsa-miR-106b-5p, such as *PAX6* and *MCL1*, using stomach, liver, and other cancer data, which could serve as endpoints for further AI-driven modeling [82]. Thus, there is a significant opportunity for future research to leverage AI to integratively combine expression datasets, predictive tools, and validated MTIs for miR-106b-5p, to identify context-specific roles across cancer types, treatment settings, and clinical outcomes.

### 3.2. Potential of AI in Identifying Biomarkers, Therapeutic Targets, and Personalized Strategies

Machine learning algorithms can be trained on large datasets of miR-106b-5p expression in various cancers, integrating both bioinformatic predictions (from TargetScan, miRDB, DIANA microT-CDS, TarBase, etc.) and experimentally validated interactions (from miRTarBase, TarBase, StarBase) to identify robust expression signatures correlated with outcomes such as overall survival, metastasis, or therapy resistance [76]. For example, combining predictive and validated target data through correlation analyses (mRNA/protein) could reduce the false positives inherent to in silico predictions alone. The example from MiR-mRNA correlations in pancreatic cancer demonstrates how pairing predicted targets with mRNA expression data helps refine candidate target genes for miR-106b-5p [81].

AI could also be utilized to predict novel target genes of miR-106b-5p by integrating gene expression data, miRNA expression profiles, and data from CLIP-Seq/degradome sequencing (as in StarBase or TarBase) to improve prediction accuracy and biological relevance. Deep-learning tools such as TargetNet, which use relaxed seed criteria and extended sequence context, represent advances in this direction [83]. By analyzing individual patient data, AI-augmented bioinformatics could help predict treatment efficacy based on miR-106b-5p expression patterns and other molecular features, facilitating the development of refined personalized medicine approaches.

### 3.3. AI-Driven Analysis of Gene Regulatory Networks Involving miR-106b-5p

Gene regulatory network approaches, combining bioinformatic and AI methods, can elucidate complex interactions of miR-106b-5p with its target mRNAs, lncRNAs, ceRNAs, and upstream regulators. Network analysis tools (for example, built into StarBase for ceRNA networks or DIANA Tools’ ceRNA/LncRNA modules) allow mapping of regulatory networks in pan-cancer settings [76,84,85,86].

**Figure 3:**
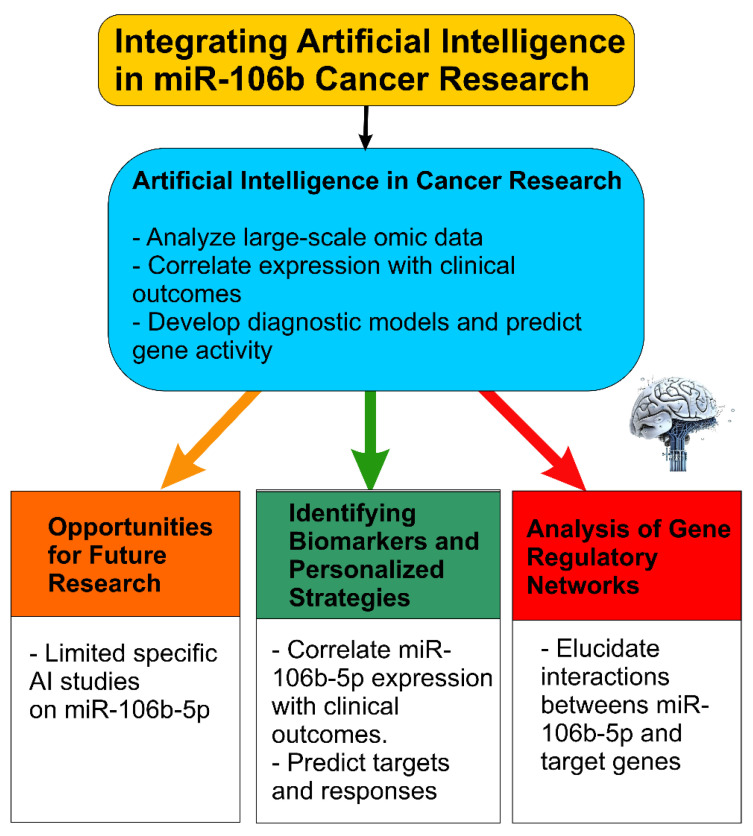
Integration of Artificial Intelligence in miR-106b-5p Cancer Research. Schematic overview illustrating the potential applications of artificial intelligence (AI) in elucidating the oncogenic and context-dependent roles of miR-106b-5p across various cancer types. AI approaches—including machine learning, deep learning, and network-based analyses—can be used to identify miR-106b-5p-related biomarkers, predict target genes, and map regulatory networks. These insights may facilitate the development of personalized treatment strategies, improve diagnostic and prognostic accuracy, and uncover novel therapeutic targets by leveraging large-scale omics datasets and histopathological image analysis. This figure has been created by MCAL and AL using CorelDRAW X8 and it is available at the UMU Institutional Repository DIGITUM [copyright Creative Commons].

AI methods, such as graph neural networks or network-based ML, could operate over these inferred networks to detect hub genes, key pathways modulated by miR-106b-5p, or condition-specific regulatory modules (e.g., in metastasis versus primary tumor, or drug-resistant vs. sensitive). Integrating multi-omics data (genomics, transcriptomics, proteomics, epigenomics) along with imaging or histopathology, when available, could provide a systems-level view of miR-106b-5p’s oncogenic or tumor-suppressive roles that vary by tissue or microenvironment (Figure 3).

Taken together, while AI tools are transforming cancer research by enabling the analysis of complex omics data, the current literature reveals limited applications of AI specifically focused on miR-106b-5p. Future work should:

- Combine predictive bioinformatic tools (TargetScan, miRDB, DIANA, StarBase) and experimentally validated databases (miRTarBase, TarBase) when building AI models, to reduce false positives.
- Use multi-omics datasets (miRNA, mRNA, lncRNA, proteome) plus clinical metadata (survival, response) to train AI/ML models for predicting biomarkers or prognostic signatures specific to miR-106b-5p.
- Apply network analysis (ceRNA, ceRNA-miRNA-mRNA, regulatory networks) with AI to identify hub genes and context-specific modules involving miR-106b-5p.
- Explore deep learning models tailored for target prediction (e.g., TargetNet) or for integrative tasks such as combining imaging + molecular data.

## 4. miR-106b-5p in Cancer Therapy-Related Cardiovascular Toxicity: A Key Mediator of Cardiac Dysfunction

**Figure 4:**
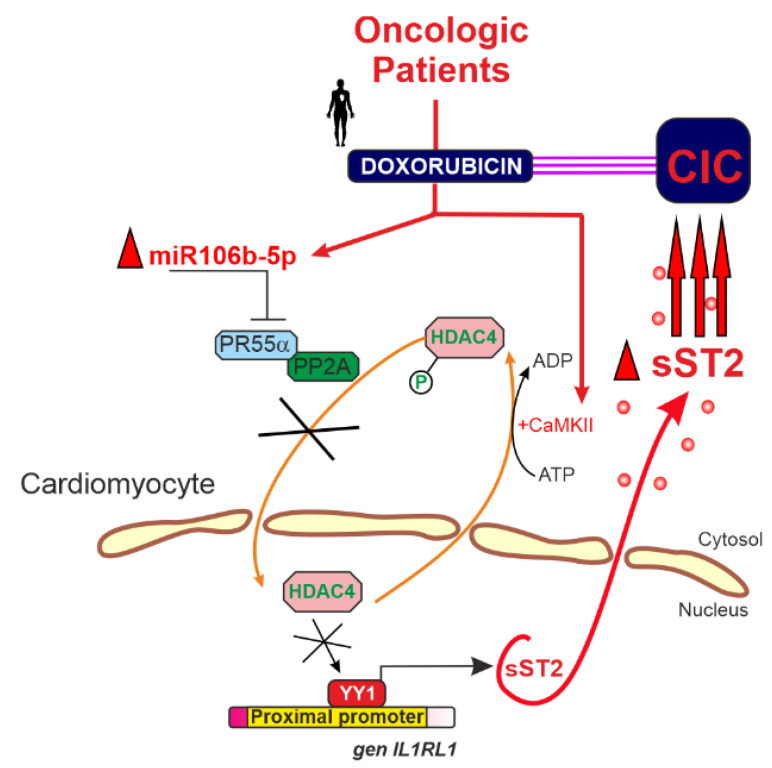
Role of miR-106b-5p in Chemotherapy-Induced Cardiotoxicity. Graphical representation of miR-106b-5p as a central modulator linking oncogenesis and anthracycline-induced cardiac dysfunction. Doxorubicin-induced upregulation of miR-106b-5p in the myocardium suppresses PR55α, reducing PP2A activity and promoting cytoplasmic HDAC4 accumulation, thereby enhancing YY1-driven sST2 expression and adverse cardiac remodeling. Concurrently, miR-106b-5p modulates oncogenic pathways including PTEN, p21/CDKN1A, PI3K/AKT, and TGF-β. Targeting miR-106b-5p with anti-miR therapy holds promise for preserving cardiac function while impeding tumor progression, supporting its translational potential as a dual-action therapeutic strategy. This figure has been created by MCAL and AL using CorelDRAW X8. The scheme is available at the UMU Institutional Repository DIGITUM, which is protected by copyright [Creative Commons]. Graphical abstract from Lax et al., 2023 [13].

The clinical utility of potent anti-tumor agents like doxorubicin is often compromised by cardiotoxic side effects that can lead to long-term cardiac dysfunction [87]. A study recently published by our group identified, for the first time, a critical role for miR-106b-5p in anthracycline-induced cardiotoxicity [13]. Using an in vivo model of cancer therapy-related cardiovascular toxicity (CTR-CVT), we demonstrated that doxorubicin treatment markedly upregulates miR-106b-5p expression in the left ventricular myocardium. Elevated levels of miR-106b-5p suppress PR55α—a key regulatory subunit of protein phosphatase 2A (PP2A)—thereby reducing the dephosphorylation of HDAC4 (Figure 4) [13]. This reduction leads to the accumulation of phosphorylated HDAC4 in the cytoplasm, which prevents its necessary nuclear translocation for repressing the transcription factor Yin Yang-1 (YY1) [13,88,89]. Unrestrained YY1 activity drives the expression of soluble ST2 (sST2), a biomarker linked to cardiac fibrosis, adverse remodeling, and poor clinical outcomes. These findings provided the rationale for developing a novel anti-miR-106b-5p molecule (protected under patent WO2023073118) that effectively inhibits miR-106b-5p function, restores PR55α levels, preserves PP2A activity, and prevents sST2-mediated deleterious cardiac remodeling [14].

Moreover, miR-106b-5p modulates key molecular pathways shared between cancer progression and cardiovascular dysfunction, including critical targets such as PTEN and p21/CDKN1A and the PI3K/AKT and TGF-βsignaling cascades [10,22,90,91]. As a central nexus driving both oncogenesis and cardiac stress, miR-106b-5p offers dual therapeutic benefits by potentially attenuating cancer progression while mitigating therapy-induced cardiac damage [14]. This comprehensive mechanistic insight paves the way for a precision medicine approach, in which elevated circulating levels of miR-106b-5p may serve as a biomarker to identify high-risk patients who could benefit from early intervention with our anti-miR therapy, ultimately accelerating clinical translation and improving both oncologic and cardiovascular outcomes.

To provide an overall view of the experimental evidence, the following summary table is included (Table 2). It outlines the study type, the experimental model used, the main findings, and the clinical or translational implications.

## 5. Clinical Implications and Future Directions

**Figure 5:**
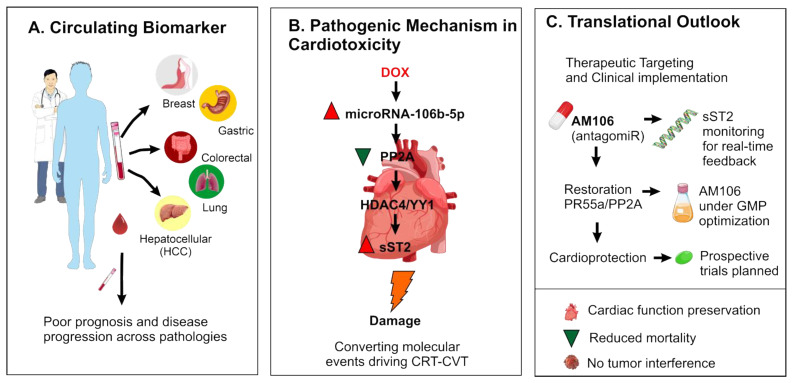
miR-106b-5p as a cross-disease biomarker and therapeutic target: pathogenic mechanisms and future directions. (**A**) Elevated circulating miR-106b-5p is associated with poor prognosis in diverse pathological contexts, including cancer (breast, prostate, hepatocellular) and systemic diseases such as coronary artery disease and pulmonary tuberculosis. (**B**) In the context of anthracycline-induced cardiotoxicity (CIC), miR-106b-5p promotes myocardial damage through suppression of PR55α and subsequent inhibition of PP2A, leading to cytoplasmic retention of phosphorylated HDAC4, disruption of HDAC4-YY1 signaling, and increased sST2 expression. (**C**) The antagomiR AM106 restores PP2A signaling, mitigates cardiac damage, and preserves cardiac function without affecting antitumor activity. sST2 may serve as a real-time biomarker of therapeutic response. AM106 is undergoing GMP optimization, with clinical trials planned. The figure has been created by MCAL and AL using CorelDRAW X8, which is protected by copyright Creative Commons.

The identification of miR-106b-5p as a circulating biomarker across multiple systemic diseases—including cancer and cardiovascular conditions—offers a valuable tool for risk stratification and early intervention (Figure 5A). In the oncology setting, elevated miR-106b-5p levels may help identify patients at increased risk of anthracycline-induced cardiotoxicity (CIC), enabling proactive clinical decision-making. Mechanistically, miR-106b-5p downregulates PR55α, leading to PP2A inhibition and pathological activation of the HDAC4–YY1–sST2 axis, which contributes to cardiac dysfunction (Figure 5B). This pathway highlights a critical molecular intersection between cancer therapy and cardiovascular injury. Therapeutically, AM106—a locked nucleic acid antagomiR developed to inhibit miR-106b-5p—has shown robust cardioprotective effects in preclinical models without compromising antitumor efficacy. These preclinical results, demonstrating robust cardioprotective effects of AM106 without compromising antitumor efficacy, support the rationale for progressing AM106 into early-phase clinical trials in patients at risk of anthracycline-induced cardiotoxicity (Figure 5C). Real-time monitoring of sST2 levels may serve as a companion biomarker to track therapeutic response and cardiac health during chemotherapy. Ongoing GMP optimization and planned prospective studies will be essential to translate these findings into clinical practice. The development of AM106 is licensed through Biocardio S.L. and protected under patent number [14], and this information is disclosed in accordance with journal policies.

To complement the visual overview provided in the figure, Table 3 summarizes the diverse roles of miR-106b-5p across oncologic and cardiac contexts. This integrated perspective highlights the complexity of its function—acting as either an oncogene or tumor suppressor depending on cellular context—and underscores its relevance as a molecular switch in key regulatory pathways. Importantly, in the setting of cardiotoxicity, miR-106b-5p exhibits a distinctly maladaptive role, further supporting its value as a dual-purpose therapeutic target.

## 6. Conclusions

This review highlights the multifaceted role of miR-106b-5p as a key regulator in both cancer and cardiotoxicity, particularly chemotherapy-induced cardiotoxicity. The evidence presented underscores its involvement in promoting malignant phenotypes in various cancers and its contribution to cardiac dysfunction through overlapping molecular mechanisms. Research on miR-106b-5p holds significant translational potential. Its presence in circulation and altered levels in cancer and cardiovascular diseases suggest it may serve as a non-invasive diagnostic and prognostic biomarker.

The development of targeted therapies, such as antagomiRs against miR-106b-5p, shows promising results in preclinical models for mitigating cardiotoxicity and potentially modulating cancer progression, supporting their evaluation in both preclinical and early clinical studies. Future research should focus on further elucidating the context-dependent functions of miR-106b-5p in different cancer subtypes and cardiovascular conditions. Identifying additional target genes and regulatory networks involving miR-106b-5p, as well as the role of other miR-106b-25 cluster members, is crucial to understanding the interplay between cancer and cardiotoxicity.

Exploring the potential of miR-106b-5p as an early biomarker for cardiotoxicity in cancer patients is of significant clinical importance. Understanding how cancer and cancer therapies influence miR-106b-5p expression in the heart, and vice versa, as well as its role in mediating communication between cancer cells and cardiomyocytes, may reveal novel therapeutic approaches.

The application of AI in analyzing the vast amounts of data related to miR-106b-5p expression and function will be crucial for accelerating discoveries and translating them into clinical benefits.

**Figure 6:**
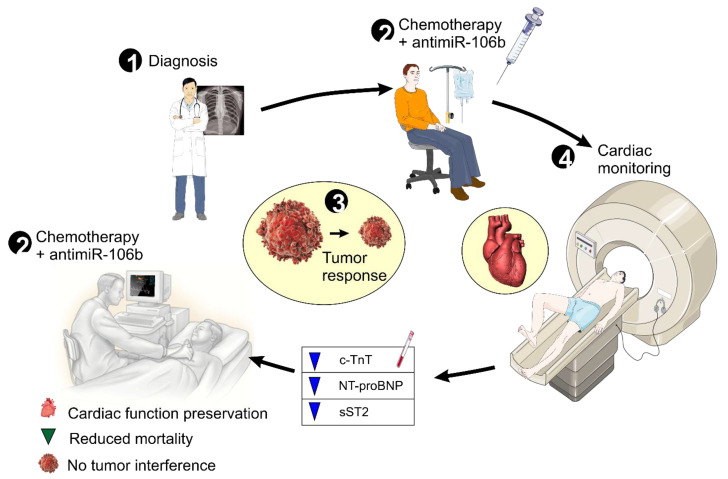
Dual-action therapeutic strategy targeting miR-106b-5p for cardio-oncology applications. This schematic represents the proposed integrative treatment approach combining conventional chemotherapy with miR-106b-5p inhibition using the antagomiR AM106. While chemotherapy targets tumor cells, it also induces miR-106b-5p expression, contributing to cardiac damage via molecular dysregulation. AM106 counteracts this by restoring PP2A function, preserving HDAC4 nuclear localization, and reducing sST2 expression, ultimately protecting the myocardium. This dual-action model aims to reduce tumor burden while simultaneously preventing chemotherapy-induced cardiotoxicity, offering a paradigm shift toward safer, more personalized cancer care. The figure has been created by MCAL and AL using CorelDRAW X8, which is available at the UMU Institutional Repository DIGITUM, copyright Creative Commons.

In conclusion, understanding the molecular links between cancer and its complications, such as cardiotoxicity, is fundamental to improving patient outcomes. miR-106b-5p emerges as a key player in this complex interaction, and further research, especially integrating AI-driven analysis and the development of targeted therapies like AM106, could lead to innovative strategies for diagnosis, prevention, and treatment of both cancer and its cardiovascular complications (Figure 6).

## Figures and Tables

**Table 1:** 106b-5p in Various Cancer Types.

Cancer Type	Role of microRNA-106b-5p	Key Target Genes	Affected Signaling Pathways	Key References
Breast Cancer	Oncogenic/Tumor Suppressor (context-dependent)	PTEN, CNN1, GAB1, GNG12, HBP1, SESN1	PI3K/AKT, Rho/ROCK1, TGF-β	[21,24,51,52]
Prostate Cancer	Oncogenic	PTEN, p21, Caspase-7, adhesion molecules	PI3K/AKT, cell cycle regulation	[53,54,55,56]
Non-Small Cell Lung Cancer (NSCLC)	Oncogenic	BTG3, PKD2, IGSF10 (context-dependent)	Proliferation, apoptosis, cisplatin resistance	[57,58,59]
Gastric Cancer	Oncogenic	SMAD7, PTEN, ALEX1	TGF-β inhibition, migration/invasion	[60,61,62]
Colorectal Cancer	Oncogenic/Tumor Suppressor (context-dependent)	FAT4, CTSA, DLC-1	Migration, invasion, metastasis, radioresistance	[63,64,65]
Hepatocellular Carcinoma	Oncogenic	PTEN, APC, BTG3, BIM	PI3K/AKT, cell cycle, EMT	[66,67,68]
Esophageal Squamous Cell Carcinoma	Oncogenic	PTEN, SMAD-7, HPGD	EMT, proliferation, invasion	[40,69]
Renal Cell Carcinoma (RCC)	Oncogenic	SETD2, CIC, Wnt inhibitors (indirect), β-catenin targets (Cyclin D1, MYC, CD44, MMP7)	Wnt/β-catenin activation, MAPK signaling, epigenetic regulation	[42,44,70]
Bladder Cancer	Prognostic biomarker/Oncogenic	TP53-related networks, cell cycle regulators	p53-like tumor biology, apoptosis, recurrence risk	[11,71,72]
Melanoma	Oncogenic	PTEN (context-dependent), EphA4	PI3K/AKT, ERK/MAPK, EMT via exosomes	[48,73,74,75]

This table summarizes the role of miR-106b-5p across different cancers, detailing whether its behavior is oncogenic or context-dependent (potential tumor suppressor effects), the key target genes, and the affected signaling pathways. This comparative overview will help us quickly appreciate the complexity of miRNA function in distinct oncological contexts.

**Table 2:** Summary of Experimental Models and Key Findings.

Study Type	Experimental Model	Major Findings	Clinical/Translational Implications	Key References
In Vitro	Cancer cell lines (breast, prostate, NSCLC)	miR-106b-5p modulates proliferation, apoptosis, migration, and invasion by targeting PTEN, BTG3, and others	Potential diagnostic and prognostic biomarker; target for therapeutic intervention	[52,92]
In Vivo	Animal models of doxorubicin-induced cardiotoxicity	Upregulation of miR-106b-5p linked to cardiac dysfunction; inhibition restored PP2A function and improved outcomes	Provides proof-of-concept for anti-miR therapy (AM106) to prevent cardiotoxicity	[13,93]
Clinical	Analysis of circulating miRNA levels in cancer patients	Elevated miR-106b-5p correlates with poor outcomes and higher risk of cardiotoxicity when using anthracyclines	Supports the use of miR-106b-5p as a stratification biomarker and guides personalized therapy strategies	[20,94]

This table provides an at-a-glance 1 summary of key experimental studies addressing miR-106b-5p, outlining the study design (in vitro/in vivo/clinical), model type, major findings, and clinical or translational implications. This summary can be used to highlight the breadth of evidence that supports the role of miR-106b-5p in cancer and cardiotoxicity.

**Table 3:** miR-106b-5p Targets and Pathways in Cancer and Cardiac Conditions.

Disease Context	miR-106b-5p Function	Key Target Genes	Affected Signaling Pathways
**Osteosarcoma**	Oncogenic	CDKN1A/p21	Cell cycle
**Non-Small Cell Lung Cancer**	Oncogenic	BTG3	Proliferation, Apoptosis
**Colorectal Cancer**	Tumor Suppressor/Oncogenic	FAT4, SLAIN2	Migration, Invasion, Metastasis
**Hepatocellular Carcinoma**	Oncogenic	PTEN, BTG3, BIM	PI3K/AKT, Proliferation, Apoptosis, Cell cycle
**Malignant Melanoma**	Oncogenic	PTEN	Akt/ERK, Cell cycle
**Prostate Cancer**	Oncogenic	PTEN, p21	Viability, Migration, Cell cycle
**Clear Cell Renal Cell Carcinoma**	Oncogenic	SETD2	Cell cycle, Apoptosis
**Breast Cancer**	Oncogenic/Tumor Suppressor	PTEN, CNN1	PI3K/AKT, Rho/ROCK1, TGF-beta
**Doxorubicin-Induced Cardiotoxicity**	Pro-cardiotoxic	FOG2, sST2	Apoptosis, Oxidative stress
**Oxidative Stress in Neonatal Mouse Cardiomyocytes**	Cardioprotective	Unknown	Antioxidant, Inflammatory

## Data Availability

The data and materials used in this review are publicly available through the referenced sources cited throughout the manuscript. All relevant datasets and materials were obtained from publicly accessible databases and repositories. No new datasets were generated or analyzed during this review. Specifically, the data from the experiments involving microRNA-106 and its link to cardiotoxicity, as well as the design and development of the patented anti-miR, are available upon reasonable request from the corresponding author. Access to certain materials may be restricted due to the proprietary nature of the patent.

## Abbreviations

3’UTR	3’ Untranslated Region
AKT	Protein kinase B
AM106	AntimiR específico contra miR-106b-5p
BCL2	B-cell lymphoma 2
BNP	Brain Natriuretic Peptide
cTnT	Cardiac Troponin T
EF	Ejection Fraction
EMT	Epithelial-to-Mesenchymal Transition
GMP	Good Manufacturing Practice
HDAC4	Histone Deacetylase 4
NT-proBNP	N-terminal pro b-type Natriuretic Peptide
PI3K	Phosphoinositide 3-Kinase
PP2A	Protein Phosphatase 2A
PR55α	Regulatory subunit of PP2A
SMAD7	Mothers against decapentaplegic homolog 7
sST2	Soluble Suppression of Tumorigenicity 2
TGF-β	Transforming Growth Factor Beta
YY1	Transcription factor Yin Yang 1
